# The broad spectrum of cancer and immunotherapy: achievements and limitations

**DOI:** 10.3389/fimmu.2025.1697505

**Published:** 2026-01-12

**Authors:** Arifa Aman, Belén Toledo, Aitor González-Titos, Manuel Picon-Ruiz, Pablo Hernández-Camarero

**Affiliations:** 1 Biopathology and Regenerative Medicine Institute (IBIMER), Centre for Biomedical Research, University of Granada, Granada, Spain; 2 ASA Research Ventures Private Limited, Lahore, Pakistan; 3 Department of Health Sciences, University of Jaén, Jaén, Spain; 4 Department of Medical Oncology, Cancer Center Amsterdam, Amsterdam University Medical Center (UMC), Vrije Universiteit University, Amsterdam, Netherlands; 5 Unit “Modeling Nature” (MNat), University of Granada, Granada, Spain; 6 Department of Human Anatomy and Embryology, Faculty of Medicine, University of Granada, Granada, Spain; 7 Instituto de Investigación Sanitaria ibs. GRANADA, Granada, Spain

**Keywords:** adoptive cell therapy, cancer immunotherapy, cancer vaccines, chimeric antigen receptor (CAR) cell, immune checkpoint inhibitors, immune resistance

## Abstract

Cancer immunotherapy has revolutionized cancer treatment over the past decades, offering renewed hope to patients with previously untreatable malignancies. This therapeutic approach could be categorized into three primary strategies: immune checkpoint blockade, adoptive cell therapy, and cancer vaccines. Immune checkpoint inhibitors have been highly successful in boosting anti-tumour immune responses by blocking the immunosuppressive signals that cancer cells exploit to evade immune surveillance, mainly that exerted by cytotoxic T lymphocytes. Adoptive cell therapy, particularly chimeric antigen receptor (CAR)-T cell therapy, involves the infusion of genetically modified cytotoxic T cells to specifically target tumour cells, showing particular efficacy in hematological malignancies. Cancer vaccines have also emerged as a promising strategy, eliciting anti-tumour responses via the patient’s own immune system. Despite these advancements, several challenges persist, particularly in the treatment of solid tumours. These include the development of tumour resistance, off-target effects that lead to adverse side effects, manufacturing complications, and variability in patient clinical outcomes. Overcoming these limitations will require further research and innovation to optimize the clinical translation of immunotherapy and broaden its application toward more personalized medicine. This review highlights the advancements and key challenges in the mentioned cancer immunotherapy strategies, with a special emphasis on the reinforcement of adaptive immune system against tumour cells. Additionally, some alternative approaches relying on the modulation of innate immune system are also summarized.

## Introduction

The link between immunity and cancer was first noted 150 years ago when erysipelas, a microbial skin condition, appeared to confer protection against cancer. Specifically, in 1893, William Coley observed improved outcomes in sarcoma patients with erysipelas, suggesting an immune-mediated response against cancer cells (1). Over a century ago, researchers proposed that the immune system continuously eliminates cancerous cells, preventing many tumours from developing (2). This concept eventually evolved into the cancer immune-surveillance hypothesis, which states that the immune system can recognize and target cancer cell antigens to suppress tumour formation (2).

**Figure 1:**
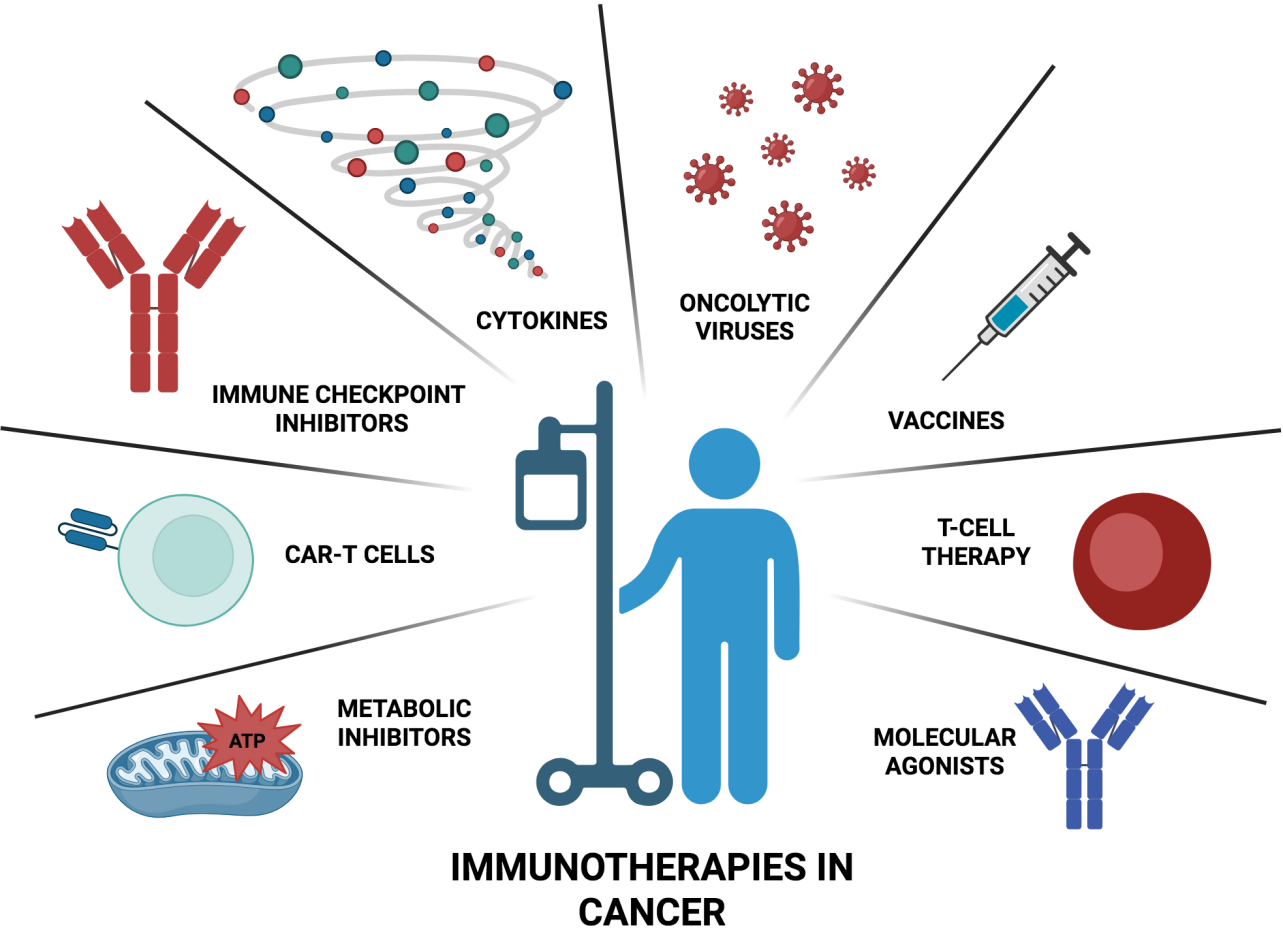
Current immunotherapy strategies in cancer. A wide range of compounds including immune-regulatory cytokines, immune checkpoint inhibitors, own immune cells, cancer vaccines, molecular agonists, metabolic inhibitors and oncolytic viruses represent some of the most widely used immunotherapies against cancer.

In recent years, cancer immunotherapy has revolutionized oncology, significantly improving the prognosis of patients with previously untreatable malignancies. Immunotherapies encompass a wide range of strategies, including the use of immune-regulatory cytokines, immune checkpoint inhibitors (ICIs), adoptive cells therapy (ACT), cancer vaccines or oncolytic viruses, all aimed to modulate the immune system for targeting and eliminating cancer cells (3) (**Figure 1**).

Among the most common ICIs, it can be mentioned antibodies targeting PD-1, PD-L1, or CTLA-4, which have yielded durable responses across a variety of malignancies such as melanoma, breast cancer or gastric cancer (4–6). These agents primarily function by releasing the “brakes” on exhausted T cells, thereby restoring their effector functions and promoting tumour eradication (**Table 1**). The success of ICIs has been underscored by their ability to induce long-term remission in a significant subset of patients, although response rates remain variable and resistance mechanisms are actively being investigated (7, 8).

**Table 1:** Main current immunotherapy approaches and properties.

Immunotherapy approach	Peptide/Molecule- or Cell-based strategy	Innate immune system modulation	Adaptive immune system modulation
Immune checkpoint blockade	Peptide/Molecule-based	anti-CD47 anti-IGSF8	anti-PD1/PD-L1 anti-CTLA4 anti-LAG3
Adoptive cell therapy	Cell-based	CAR-Macrophages CAR-NK	CAR-T cells TCR-engineered T cells
Cancer vaccines	Both	Toll-like Receptors (TLRs) Bacillus Calmette-Guérin	Peptides- DCs- Whole Tumour Cells- IPSCs- In Situ- Nucleotides- based vaccines
Cytokine Therapy	Peptide/Molecule-based strategy	GM-CSF IFN-α/-β	IL-2, IL-12 IFN-α/-β
Oncolytic Virus Therapy	Cell-based strategy	TLRs RIG-I Inflammatory Response	Antigen Release and Presentation IL-12 GM-CSF

ACT, particularly chimeric antigen receptor (CAR)-T cells and TCR-engineered T cells, represents another frontier in cancer immunotherapy. CAR-T cell therapies, exemplified by treatments targeting CD19 in hematologic malignancies, have demonstrated remarkable clinical efficacy, achieving high remission rates in refractory leukaemia and lymphomas (9, 10). Advances in gene editing and cell manufacturing are expanding the applicability of these therapies to solid tumours, where challenges such as immune suppression, antigen heterogeneity, and tumour microenvironment (TME)-related barriers are being actively addressed (11). Moreover, TCR-engineered T cells allow for recognition of intracellular tumour antigens presented by Major Histocompatibility Complex (MHC) molecules, broadening the spectrum of targetable tumour-associated antigens (12) (**Table 1**).

Beyond adaptive immune modulation, innate immune system pathways are being exploited to stimulate anti-tumour immunity. Toll-like receptors (TLRs), a critical component of the innate immune response, recognize pathogen-associated molecular patterns and facilitate the activation of dendritic cells, macrophages, and natural killer (NK) cells (13). TLR agonists such as CpG oligodeoxynucleotides (TLR9 agonists) and monophosphoryl lipid A (TLR4 agonist) have been investigated as adjuvants in cancer vaccines and as monotherapies to enhance antigen presentation and cytokine production, with preclinical studies showing robust activation of anti-tumour immune responses (14, 15). Additionally, modulation of other innate pathways, including RIG-I agonists and inflammasome activation (NLRP3 inflammasome), further amplifies immune activation and fosters a pro-inflammatory TME (16, 17) (**Table 1**).

Cytokine therapy remains a cornerstone of immunomodulation, with interleukins such as IL-2 and IL-12 demonstrating potent activation and proliferation of cytotoxic T lymphocytes and NK cells. High-dose IL-2 has historically achieved durable remissions in some metastatic melanoma and renal cell carcinoma cases, although toxicity limits its use (18). More recently, novel cytokine formulations and targeted delivery strategies aim to enhance efficacy while minimizing adverse effects. Interferons, especially IFN-α, have also been employed to boost antigen presentation and tumour immune cells infiltration, with ongoing efforts to optimize their clinical utility (19) (**Table 1**).

Oncolytic virus therapy represents an innovative approach that combines direct tumour cell lysis with immune stimulation (20). Genetically engineered viruses such as talimogene laherparepvec (T-VEC), a modified HSV-1, have demonstrated clinical benefits in melanoma by inducing immunogenic cell death and promoting systemic anti-tumour immunity (21). These viruses selectively replicate in tumour cells, releasing tumour-associated antigens and triggering innate immune pathways, including TLRs and RIG-I/MDA5, thereby creating an inflamed TME conducive to subsequent immune checkpoint blockade (22) (**Table 1**). Together, these diverse immunotherapeutic strategies are converging toward a more personalized and effective approach to cancer treatment. They harness both innate and adaptive immunity, exploit tumour-specific vulnerabilities, and aim to overcome mechanisms of immune evasion, marking an era of unprecedented progress and hope in oncology (20).

However, mainly solid tumours exemplified by triple-negative breast cancer frequently display leaky and structurally abnormal vasculature along with high interstitial pressures, which contribute to increased resistance to immunotherapy-based strategies like adoptive T cells transfer (23). Moreover, the surrounding TME can boost cancer immune evasion and reduce immunotherapy effectiveness. In this context, it has been described that the specific stromal-myeloid ecosystem of peritoneal metastases, but not that from primary gastric cancers, may be the major mediator of immune checkpoint blockade-based treatment failure (24). Similarly, the presence of intrinsic genomic mutations can reduce sensitivity of own tumour cells to immune surveillance, being SMARCA4 mutation in lung cancer a representative example (25). In this sense, cancerous cells (i.e. metastatic melanoma) can also develop immune tolerance during tumour progression through the acquisition of new particular mutations in specific genes such as SEC24C and SEC24D, making them resistant to ICIs even after being initially vulnerable (26).

This review focuses on the advancements in three main cancer immunotherapy-based strategies: immune checkpoint blockade, adoptive immune cells transfer and cancer vaccines, with a special emphasis on the reinforcement of adaptive immune system against tumour cells. Additionally, the main hurdles related to the broad clinical implementation of immunotherapies are also discussed, including the contribution of immunosuppressive TME, the development of tumour immune resistance, the off-target effects that lead to adverse side effects, the existence of manufacturing complications, or the variability in patient clinical outcomes. Complementary, some alternative approaches relying on the modulation of innate immune system are also briefly introduced.

## Immune checkpoint inhibitors

A key function of the immune system is its ability to discriminate body’s healthy cells (needed for preservation) from those deemed abnormal or foreign (targeted for eradication), such as cancer cells and pathogens. A critical aspect of such choice relies on the presentation of “aberrant” antigens on the surface of cancerous cells, which signals their foreign nature to immune cells (27). Effector T cells, once activated upon antigen recognition, proliferate and differentiate to destroy cancer cells, but they must be inactivated afterward to prevent autoimmune reactions. This regulation is mediated by “checkpoint” proteins (both activators and inhibitors), which act as switches that control the intensity of the immune response (28).

Several inhibitory immune checkpoints, such as PD-1 (Programmed Death-1), CTLA-4 (Cytotoxic T-Lymphocyte-Associated Protein 4) (29, 30), and LAG-3 (Lymphocyte Activation Gene-3) (31), prevent the referred excessive immune activation against healthy tissues. However, cancer cells can exploit these inhibitory checkpoints to evade immune surveillance. For instance, in hepatocellular carcinoma, the PRDM1-USP22-SPI1 axis has been identified as a key pathway that upregulates PD-L1 (a PD-1 ligand) by cancerous cells, contributing to T cell exhaustion through the activation of its PD-1 receptor (32). The tumour stroma can also contribute to immune escape of cancerous cells (33). In breast cancer, high stromal dihydrolipoyl dehydrogenase (DLD) expression correlates with increased PD-L1 expression, which is also associated with enhanced tumour cell migration, invasion and proliferation (34). These findings underscore the complex interplay between the immune system, tumour cells and the TME, specially in solid malignancies.

A wide range of compounds has been engineered to target/block inhibitory checkpoint proteins, known as immune checkpoint inhibitors (ICIs). Notably, these inhibitors used in the therapeutic strategy termed “immune checkpoint blockade”, do not directly kill cancer cells like traditional anticancer treatments, reinforcing their innovative potential (35). As a representative example of ICIs, the PD-1/PD-L1 axis blockade rapidly became a popular approach. AUNP-12, a peptide patented in 2014, was the first peptide-based inhibitor targeting the PD-1/PD-L1 interaction, showing promising results to reduce tumour proliferation and metastasis in preclinical animal studies (36). Similarly, other peptide-based inhibitors, including those with 7–8 amino acids structure and cyclopeptide derivatives, have also demonstrated significant anti-tumour activity, particularly in melanoma models (37). Regarding small molecule-based inhibitors, BMS-8 and BMS-202 act through the induction of PD-L1 dimerization. These small molecules offer advantages such as enhanced tissue penetration, the ability to target intracellular pathways and the synergism with other inhibitors targeting epigenetic or metabolic pathways, thus amplifying the overall anti-tumour immune response (38).

**Figure 2:**
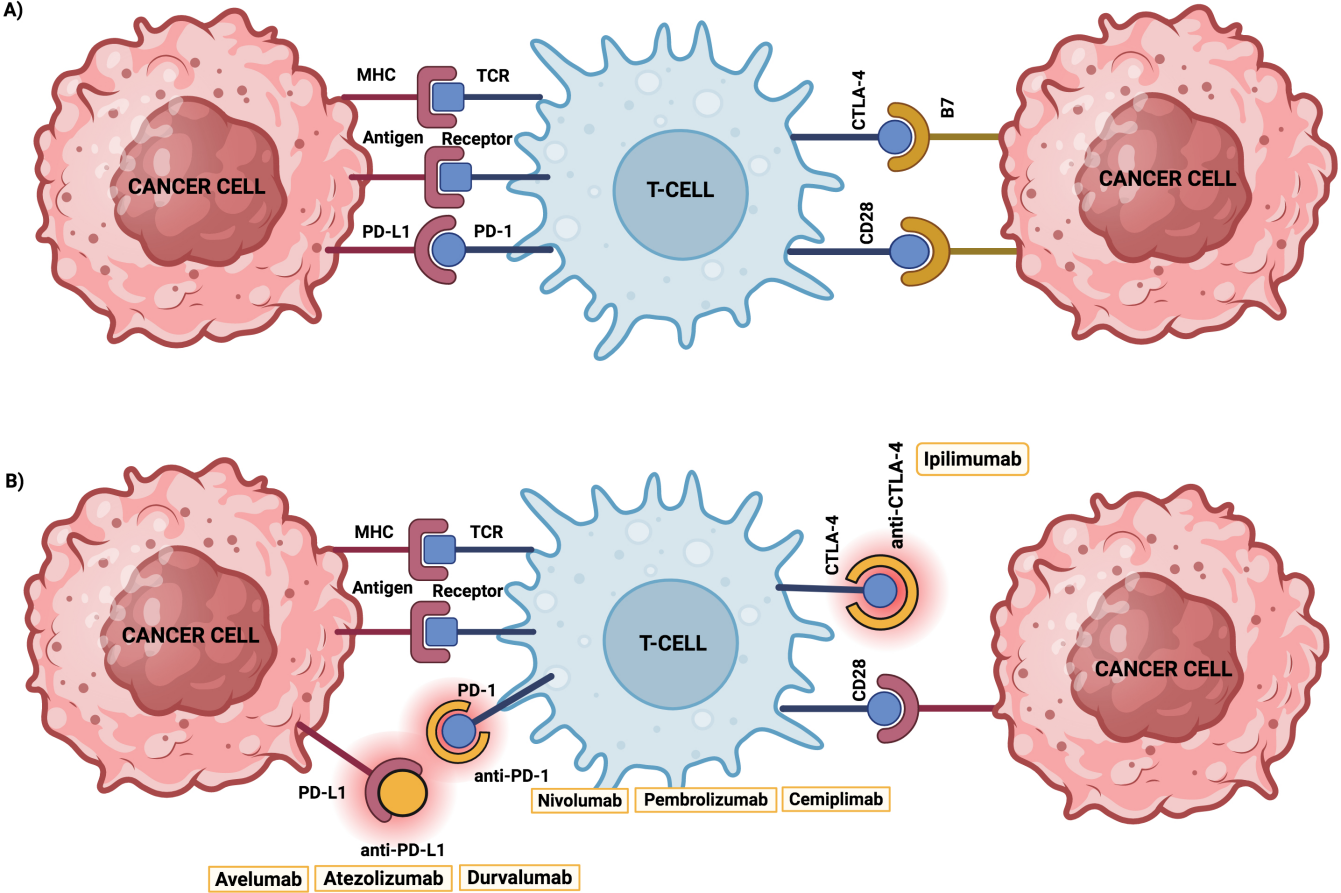
Inhibition of PD-1 and CTLA-4 as representative examples of inhibitory immune checkpoints blockade. **(A)** Tumour cells overexpress PD-L1 ligand and CTLA-4 ligand, called B7, to target and suppress T cell’s activation, leading to its exhaustion and cancer immune escape. **(B)** Several compounds, including monoclonal antibodies, have been engineered to target these inhibitory checkpoints receptors/ligands to overcome such immune-resistance cancer mechanisms.

Antibody-based ICIs have garnered special attention in the clinical setting. Sotigalimab, combined with nivolumab, has shown significant activity in overcoming anti-PD-1 resistance, achieving a 15% objective response rate in advanced and metastatic melanoma (39). Pembrolizumab treatment, an IgG4 antibody that blocks PD-1, demonstrated a 65.8% objective response rate in combination with cabozantinib in a Phase I/II trial for metastatic renal cell carcinoma (40). Additionally, cemiplimab, administrated with Q4W dosing, showed effective, rapid and durable responses with an acceptable safety profile in the treatment of advanced cutaneous squamous cell carcinoma (41). Atezolizumab was evaluated in combination with derazantinib in a Phase Ib/II trial about metastatic urothelial cancer with FGFR1–3 genetic aberrations, revealing limited efficacy but good tolerability (42). Durvalumab combined with ceralasertib showed promising clinical benefits in immunotherapy-refractory of non-small cell lung cancer, particularly in patients with Ataxia-Telangiectasia Mutated (ATM) alterations (43). Furthermore, avelumab demonstrated significant long-term overall survival benefits as a first-line treatment for metastatic Merkel cell carcinoma, establishing it as a preferred therapy over traditional chemotherapy (44) (**Figure 2**).

Despite their initial promise, ICIs still face challenges such as limited efficacy mainly in solid tumours. Notably, the CheckMate 650 trial evaluated two dual-ICB regimens combining nivolumab and ipilimumab: Arm A (nivolumab 1 mg/kg + ipilimumab 3 mg/kg every 3 weeks for four cycles, followed by nivolumab 480 mg every 4 weeks) in chemotherapy-naïve metastatic castration-resistant prostate cancer (mCRPC), and Arm B (nivolumab 3 mg/kg + ipilimumab 1 mg/kg every 3 weeks for four cycles, followed by nivolumab 480 mg every 4 weeks) in post-chemotherapy patients. The trial reported modest antitumour activity, with objective response rates of 25% in Arm A and 10% in Arm B. However, both regimens were associated with substantial toxicity, with grade ≥ 3 treatment-related adverse events occurring in 42% and 53% of patients in Arms A and B respectively, underscoring the need for dose and schedule optimization for combination checkpoint blockade in prostate cancer (45). Considering such limitations/variability in the clinical outcomes, identifying patients who may benefit from the use of ICIs is challenging and exhibits a vital importance. Factors like the expression levels of immune checkpoints or microsatellite instability (MSI) profiles, which alters tumour mutational burden (TMB), have been proposed as biomarkers for ICIs effectiveness. In fact, cancers with low TMB may not generate sufficient neoantigens to be recognized by the host immune system and thus ICIs may be less effective (46). For instance, the treatment of pancreatic cancer (PC) with ICIs revealed better outcomes in patients with high-TMB caused by heightened DNA mismatch repair gene mutations, while KRAS mutations seen in low-TMB patients may be associated to limited ICIs outcomes (47). Similarly, high TMB and MSI are predictive of ICIs sensitivity in melanoma and lung cancer (48). Nevertheless, they are not universally reliable biomarkers as other factors related to the complexity of the TME, such as the hypoxic conditions, its fibrotic and dense extracellular matrix (which hinders the penetration of therapeutic drugs), and the presence of immunosuppressive cells (e.g., regulatory T cells [Treg], myeloid-derived suppressor cells [MDSCs]) may also hinder ICIs effectiveness (49).

Moreover, differences in genetic backgrounds and epigenetic modifications can also influence how patients respond to ICIs (50). In particular, epigenetic modifications like DNA methylation and histone modification can silence immune-related genes. For example, the combination of histone deacetylase (HDAC) inhibitors with ICIs are required in colorectal carcinoma (51). Additionally, emerging evidences suggest that diet and gut microbiota composition could impact ICI efficacy, contributing to increase variability in patient responses (52). In fact, manipulating the gut microbiome through probiotics or fecal microbiota transplantation could potentially enhance the response rates of immunotherapies in non- or low-responding patients (53). Besides, patients with autoimmune diseases, chronic infections or those under immunosuppressive treatments may have impaired responses to ICIs. For example, a study found that early-Phase trials with PD-1/PD-L1 inhibitors overestimated ICIs efficacy compared to Phase III trials, largely due to the exclusion of patients with autoimmune diseases thus underscoring the need for more accurate early-Phase trial designs (54).

On the other hand, ICIs can cause immune-related adverse events (IRAEs) affecting various organs, highlighting the skin (psoriasis) (55), gastrointestinal tract (56), liver (57), endocrine glands (58) and lungs (59). Managing these toxicities is complex and may require discontinuation of therapy to avoid further worsening patients quality of life, thus limiting therapy efficacy (60). Notably, ICIs can cause various endocrine IRAEs, including thyroid dysfunction and immune-related hypophysitis (IH), the latter gaining increasing attention due to its complexity and non-specific clinical manifestations, often resulting in misdiagnosis and worse prognosis. In fact, a study of 40 melanoma patients with ICI-induced hypophysitis revealed that clinical side effects varied significantly between ipilimumab and anti-PD-1 treatments, underscoring the need to monitor specific markers based on the ICI regimen (61). In addition, Prolonged immune activation derived from long-term ICIs therapy can cause chronic inflammation and tissue damage, exemplified by a case of rapid-onset inflammatory arthritis post-pembrolizumab dosage (62).

**Figure 3:**
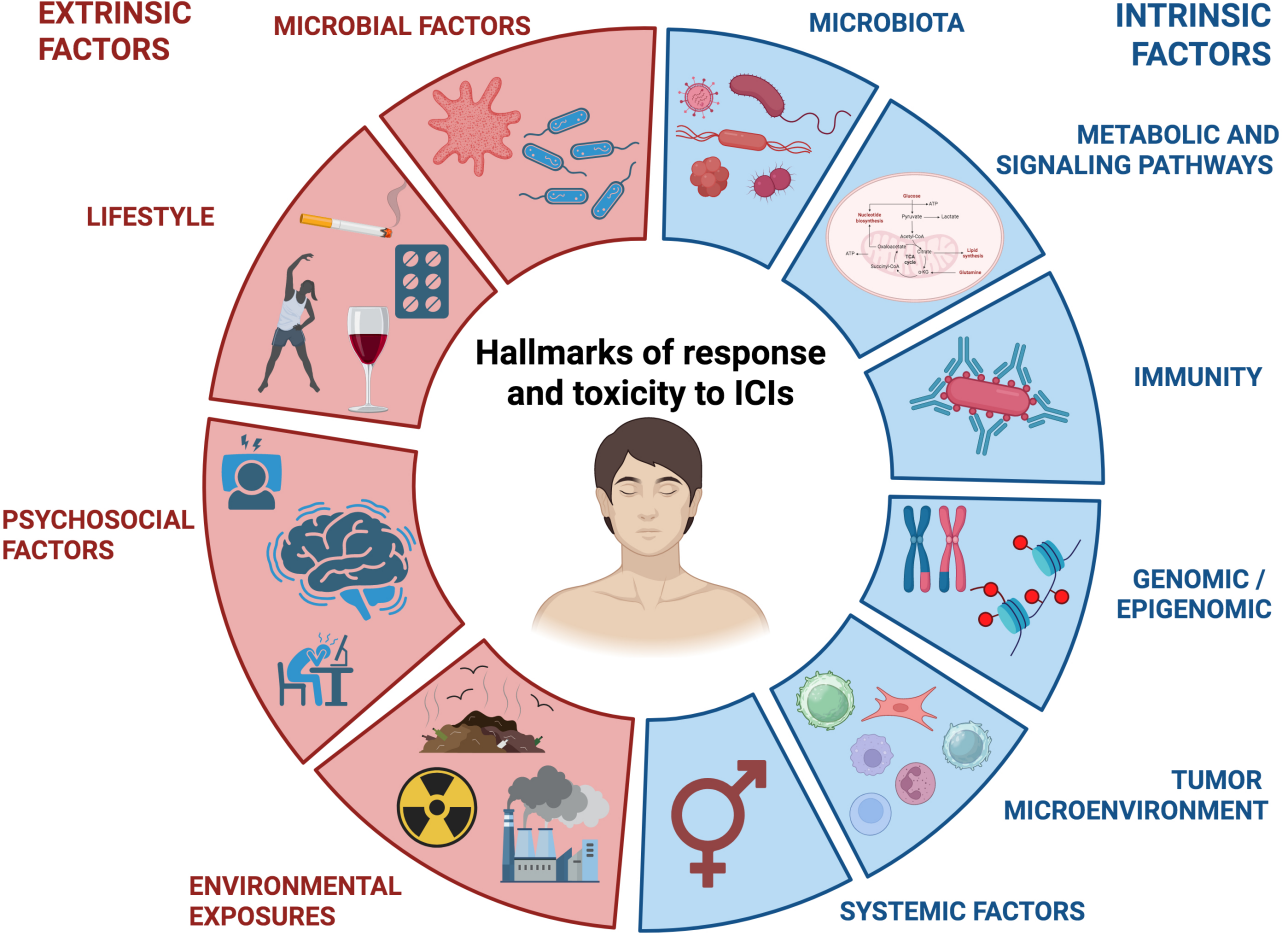
Hallmarks of response and resistance to ICIs treatments. Extrinsic (red) and intrinsic factors (blue) that affect the development of ICIs resistance are evaluated and classified.

In a different line, patients that initially respond to ICIs can develop resistance over time. For example, tumours can evade immune detection (thus bypassing ICIs regimens) through acquiring particular mutations in antigen presentation mechanisms, including MHC (63), B2M (64) and TAP genes (65). Parallelly, the dysregulation of several molecular pathways can also alter ICIs response, including the overactivation of ERK signaling in melanoma (66) or the deficiency in STING pathway in colon cancer. Indeed, intra-tumoural administration of STING agonists, often combined with systemic ICIs, has shown promise in enhancing anti-tumour immunity and overcoming resistance (67) (**Figure 3**).

## Emerging immunotherapeautic approaches targeting innate immune system, glycol checkpoints, multi-specific antibodies and chekomine

It is noteworthy to briefly highlight the existence of immunotherapies directed against innate immune system. For instance, CD47 has been presented as an innate immune checkpoint expressed by cancerous cells in order to avoid their elimination by macrophage phagocytosis. Its blockade could raise as a novel ICI-based approach against EGFR-mutated non-small cell lung cancer with resistance to conventional anti-PD-1/PD-L1 immunotherapies (68). Similarly, it has been documented that malignant cells from several tumour types like melanoma, breast, lung and colon cancer can bypass their eradication by NK cells through the upregulation of IGSF8 (69). Specifically, this article revealed that IGSF8 binds to KIR3DL2 on NK cells surface, inhibiting their degranulation and cytotoxic capacity, and highlights the therapeutic potential of IGSF8 blockade as a monotherapy or in combination with anti-PD-1 ICIs.

Similarly, Siglec–sialic acid interactions represent a novel class of immune checkpoints known as “glyco-checkpoints”), whose blockade may be complementary to classical PD-1/PD-L1 or CTLA-4 inhibition. Indeed, aberrant sialylation on tumour cells surface constitutes a mechanism of immune evasion by engaging inhibitory Siglec receptors (e.g., Siglec-7, Siglec-9, Siglec-15) on immune cells like tumour-associated myeloid cells, NK cells and subsets of T cells (70). In breast cancer-derived bone metastasis models, blockade of the Siglec-15/sialic acid axis reversed osteoclastogenesis, impaired tumour-induced immunosuppression and restored cytotoxic T cells activity, suggesting a therapeutic window for targeting glyco-checkpoints in metastatic niches (71). In prostate cancer models, blockade of Siglec-7/9-sialoglycan interactions enhanced immune cells infiltration and inhibited tumour growth, suggesting a broader applicability beyond Siglec-15 (72).

Additionally, other antibodies-based strategies relying on targeting innate immune cells receptors could be CSF1/CSF1R blockade in TAMs, with the objective of reducing solid TME immunosuppressive potential. For instance, the blockade of CSF1R (using the monoclonal antibody AMG820) in combination with PD1 blocking antibody (Pembrolizumab) has been tested in clinical trials with colorectal cancer patients, although exhibiting a modest objective response rate of 6% (73). Preclinical studies in colorectal cancer have achieved increased anti-tumour efficacy with CSF1R inhibitory small molecule PX17 compared to AMG820. Its more pronounced anti-tumour potential relied on TAMs reprogramming (rather than eradication) from pro-tumorigenic M2 into tumour-restricting M1 phenotype, which enhanced the cytotoxic CD8+ T cells response, reduced immunosuppressive cells (Tregs and MDSCs) infiltration and improved anti-tumour efficiency of PD1 blockade (74).

Furthermore, the *in vivo* efficiency of agonistic antibodies targeting CD40, to induce the activation of antigen presenting cells and enhance cytotoxic T cells response, has been noted to be dependent on the selective crosslinking of the fragment crystallizable (Fc) gamma receptor IIB (FcγRIIB) through a Fc domain located on the own agonistic antibodies. Such Fc domain allows CD40 receptor trimerization with the objective of enhancing downstream signaling within immune cells to promote their activation (75). Indeed, a phase I clinical trial enrolling patients with locally advanced or metastatic melanoma and breast cancer highlighted the importance of optimizing Fc portion of CD40 agonistic antibodies in order to achieve meaningful clinical anti-tumour effects, and the use of species-matched Fc receptor models to accurately estimate appropriate drug dosage (76).

Moreover, the use of multi-specific antibodies, able to simultaneously target two or more epitopes, represents a popular immunotherapy-based approach. For instance, it has been explored the clinical use of SL-172154, an antibody capable of simultaneously blocking CD47/SIRPα pathway along with activating CD40 receptor, to treat platinum-resistant ovarian cancer patients through energizing antigen presenting cells, B lymphocytes and cytotoxic T cells response (77). Interestingly, such antibody contains an hexameric CD40l domain to enhance CD40 trimerization and activation, which allows silencing the Fc domain in order to mitigate side effects derived from Fc-FcγRIIB interaction (77).

Another strategy focuses on simultaneously engaging specific tumour-associated antigen(s) along with immune cells receptor(s) with the objective of redirecting immune effector cells against malignant ones, particularly those belonging to haematological tumours (78). A range of bi-specific antibodies (BSABs) are being tested under clinical trials, for instance Blinatumomab (targeting CD19 tumour-associated antigen and CD3 on immune effector cells) in a phase 1/2 trial which revealed significant tumour remission in patients with refractory or relapsed B-cell acute lymphoblastic leukaemia (79). Tri-specific antibodies (TSABs) embody next-generation which can target a broader spectrum of tumour-associated antigens or an additional immune co-stimulator with improved potential to activate tumour immune surveillance, although the majority of them are still in preclinical or early-phase clinical evaluation (78). To note, Ramantamig (simultaneously targeting BCMA, GPRC5D tumour-associated antigens along with CD3 receptor on effector T-cells) displayed enhanced anti-tumour activity against multiple myeloma in *in vitro*, *ex vivo* and *in vivo* settings (80).

Nevertheless, some limitations need to be further addressed, including the emergence of side effects like cytokine release syndrome, neurotoxicity, haematological damage, increased susceptibility to infections, and the presence of drug resistances related to specific cancer mutations, antigen loss, elevated soluble antigens or the TME immunosuppressive potential (78). Interestingly, an alternative and increasingly explored approach to overcome host T cell exhaustion is based on simultaneously engaging innate immune cells activators, like CD16 in natural killer cells and macrophages, along with tumour-related antigens such as HER2 in ovarian and breast cancer cells (81).

On the other hand, chemokines and their receptors (e.g., CXCL12/CXCR4 axis) can play an important role in tumour biology, promoting metastasis, therapy resistance and shaping the immunosuppressive TME by enhancing inhibitory immune cells (Tregs, MDSCs) recruitment/infiltration (82). Preclinical studies have confirmed that CXCR4 blockade can reduce Tregs and MDSCs infiltration and enhance antitumour immunity across multiple tumour types (83). Thus, CXCR4 blockade has been further explored as an attractive drug target to synergize with traditional immunotherapeutic agents, particularly in immunologically ‘cold’ or immune-excluded tumours. In fact, a phase IIa clinical trial utilized BL-8040 (a CXCR4 antagonist) in combination with Pembrolizumab to treat metastatic, chemotherapy-resistant pancreatic cancer patients, which demonstrated increased CD8+ T cells infiltration, decreased amount of MDSCs and Tregs and achieved disease-control in a substantial fraction of individuals (84). However, the chemokine network is highly redundant and pleiotropic, thus interventions may inadvertently block beneficial immune cells recruitment or normal immune surveillance. Furthermore, tumour heterogeneity and tumour compensatory mechanisms can limit long-term efficacy (82, 84).

## Adoptive cell therapy

**Figure 4:**
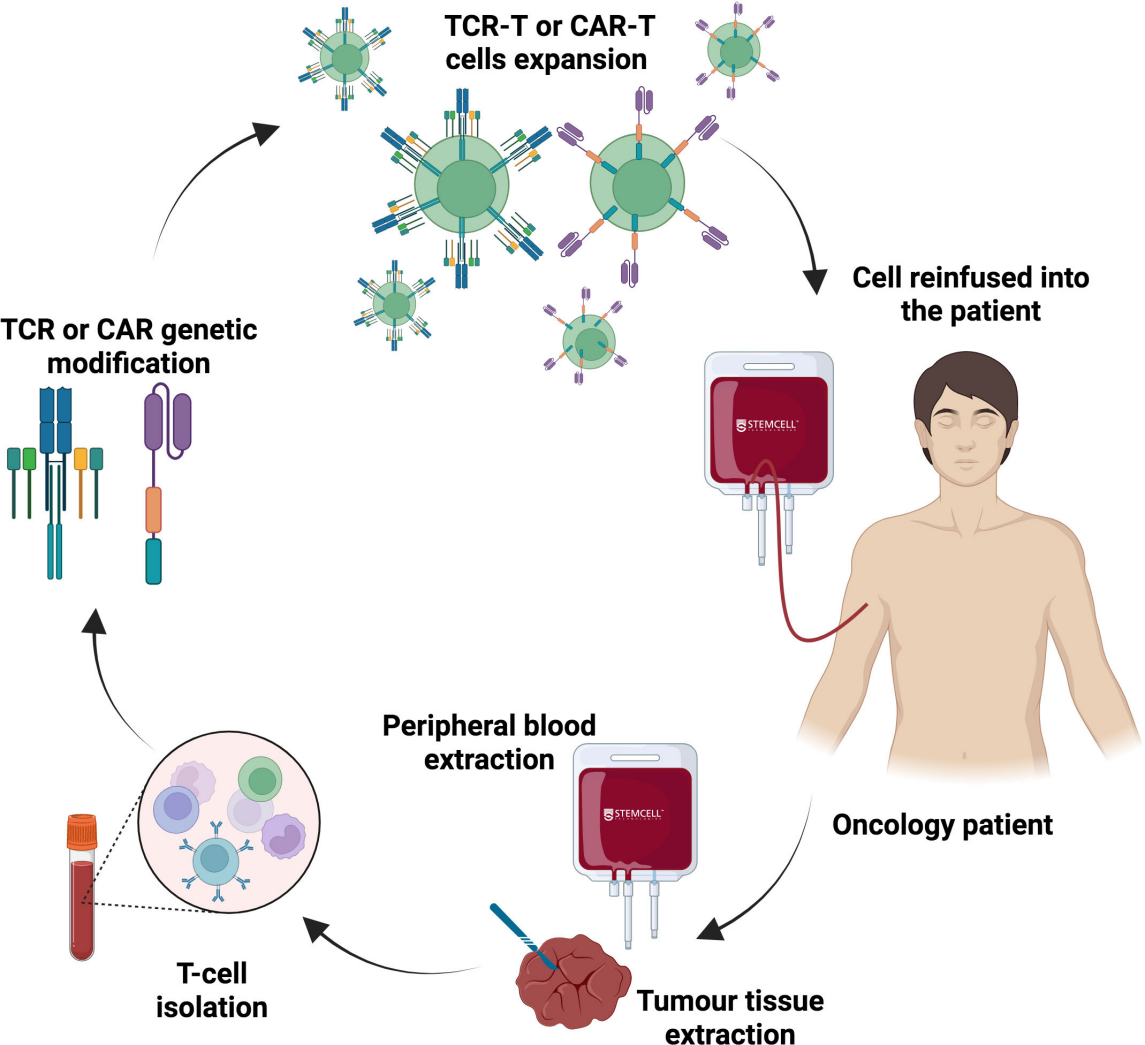
Adoptive T cell therapy: chimeric antigen receptor (CAR)-T cells and T cell receptor (TCR)-engineered T cells. T cell therapy represents a personalized treatment that uses patients’ own T cells, which are genetically modified in the laboratory. T cells are extracted from patients’ peripheral blood or tumour biopsies. Once they are recollected, T cells are genetically modified and equipped with special CARs or an engineered TCR that enable them to identify specific antigens on the surface of cancer cells. After modification, these engineered T cells are propagated and checked in the lab. When a sufficient number of CAR/TCR-T cells are generated, they are reintroduced into the patient’s body to seek out and eradicate cancer cells with the targeted antigen. These cells are designed to continue dividing in the patient’s body, using their engineered receptors to locate and eliminate any remaining cancer cells displaying the referred antigen.

Unlike traditional anticancer treatments, which often have broad side effects on healthy tissues, Adoptive T Cell Therapy (ACT) offers a more targeted and personalized approach. In fact, ACT allows customization based on the individual’s genetic and immune profiles. Briefly, ACT involves collecting autologous cells, like cytotoxic T cells, from the patient’s peripheral blood or tumour tissue. These cells are then genetically modified or activated to specifically recognize and attack cancer cells and expanded *ex vivo*. Finally, they are reinfused into the oncological patient (85). Compared to other immunotherapies, which strongly rely on the host’s immune profiles (i.e. ICIs), ACT offers advantages such as sufficient cells quantities, modifiable functions, durable responses and reduction of individuals differences (86). Currently, genetic engineering has mainly improved two key therapeutic T-cell technologies that include chimeric antigen receptor (CAR)-T cells and T cell receptor (TCR)-engineered T cells. As an example of CAR-T cells therapy, it has been observed that case-by-case CAR-T cells optimization, in order to direct them against novel antigens like VHH, may be crucial for improving the management of malignancies such as multiple myeloma (87). On the other hand, a study of TCR-engineered T cells targeting NY-ESO-1 in melanoma revealed promising results for cancer immunotherapy (88) (**Figure 4**).

### Chimeric antigen receptor T-cell therapy

**Figure 5:**
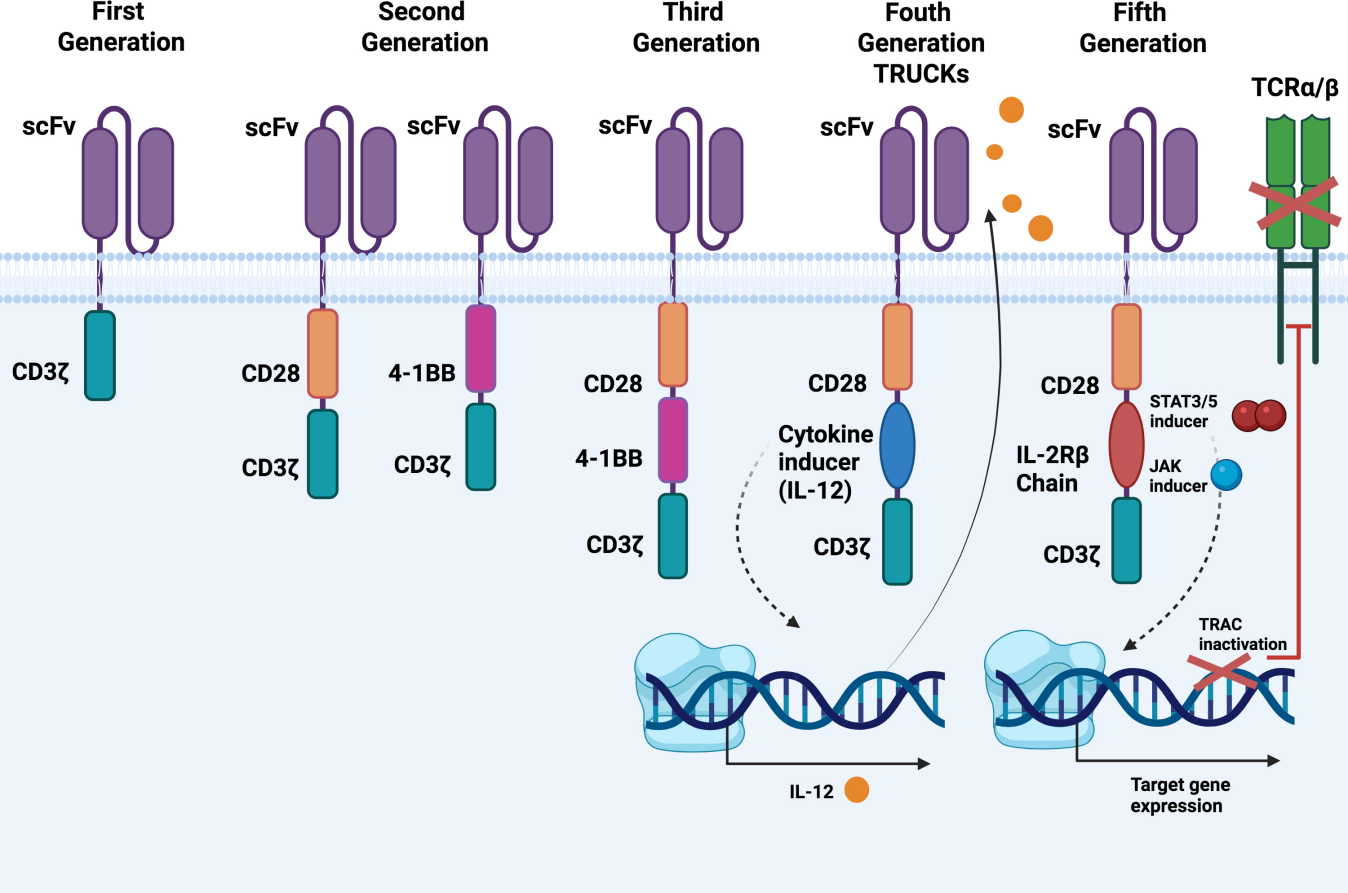
CAR-T classification according to its structure. 5 main generations of CAR-T can be identified: first-generation CARs resembled natural T-cell receptors with an extracellular scFv and an intracellular CD3ζ activation domain. The second-generation CAR-T cells incorporate a co-stimulatory molecule, such as CD28 or 4-1BB (CD137), to the intracellular domain. Third-generation CAR-T cells advanced this approach by incorporating multiple co-stimulatory signaling domains. Later generations introduced changes to transcription factor genes or added other co-stimulatory ligands (i.e. modifying scFv with short linkers or adjustments to intracellular signaling through alternative co-stimulatory domain constructs).

Several types of CAR-T cells have been authorized for clinical use to treat, primarily, hematologic malignancies. For instance, Kymriah (Tisagenlecleucel) is approved against B-cell acute lymphoblastic leukemia (89). Similarly, it has been reported the use of Yescarta (Axicabtagene Ciloleucel) against large B-cell lymphoma (90). Additional examples could be the management of refractory acute lymphoblastic leukemia (ALL) (91) or diffuse large B-cell lymphoma (DLBCL) (92). It should be highlighted that the FDA has approved multiple CAR-T cells products to target high CD19 marker expression for the treatment of both ALL and DLBCL (93). The engineering technologies of T cells are continuously improving through distinct generations (currently five main generations based on their structural evolution). The first-generation mimics natural TCR with an extracellular single-chain variable fragment (scFv) and an intracellular activation domain (CD3ζ). However, they suffer from rapid cell depletion and insufficient cytokine secretion, limiting their anti-tumour efficacy (94). The second-generation CAR-T cells were improved by adding a co-stimulatory molecule such as CD28 or 4-1BB (CD137) to the intracellular region, enhancing T cell activation and persistence. This generation forms the basis of currently FDA-approved CAR-T therapies, exemplified by “KTE-C19” (axicabtagene ciloleucel) targeting CD19 (approved under the brand name Yescarta for treating certain types of lymphomas) (95). Similarly, Second-generation anti-MSLN CAR-T cells showed promising anti-tumour effects and potential clinical value in treating ovarian cancer (96). Third-generation CAR-T cells further improved this therapeutic strategy by incorporating multiple co-stimulatory signaling domains, thereby enhancing CAR-T cell proliferation, survival, and cytokine secretion, exemplified by those incorporating both CD28 and 4-1BB costimulatory domains (97). Subsequent generations have introduced modifications to transcription factor genes or additional co-stimulatory ligands. In fact, modifications with short scFv linkers can enhance CAR-T receptor homodimerization and anti-tumour efficacy against multiple myeloma (98). On the other hand, adjusting intracellular signaling by other methodologies, like the use of alternative co-stimulatory domain constructs, has also been evaluated. B7-H3.TMIζ CAR-T cells are an example, being based on TMIGD2 co-stimulation, revealing robust anti-cancer cytotoxicity and reducing off-target side effects in glioblastoma models (99) (**Figure 5**).

An alternative therapeutic approach is the simultaneous targeting of both cancer and stromal cells within the TME. A study reported CAR-T cells engineered to target both mesothelin (a cancer-related antigen) and fibroblast activation protein (FAP) which effectively promotes the elimination of PC cells and cancer-associated fibroblasts in various patient-derived models, showing promise as a treatment option for immunotherapy-resistant solid tumours (100). However, there exist different factors contributing to the suppression of CAR-T cells related to the own TME of solid tumours, including the secretion of inhibitory immune checkpoint molecules and inhibitory cytokines by immunosuppressive stromal cells, the hypoxic conditions or the physical barrier established by the dense/fibrotic stroma. These conditions not only impair CAR-T cell infiltration into tumours but also reduce their activity upon reaching the target cells (101). For instance, it has been observed that PD-1 receptor inhibition selectively enhanced functionality and anti-tumour efficacy of low affinity HER2-28Z CAR-T cells in ovarian cancer, reducing the immunosuppressive potential of the TME (102). Furthermore, it has been observed that injection of biodegradable scaffold can enhance CAR-T cell therapy’s effectiveness by creating an alternative microenvironment that may improve tumour infiltration of T-cells, their expansion, differentiation and antitumour activity in mice models (103). Interestingly, the use of “switch” receptors has also proved their efficacy in order to convert inhibitory signals derived from the TME into stimulatory ones (104).

In addition, cancer antigen heterogeneity may represent a significant hurdle for CAR-T cells-based therapies. To combat antigen escape carried out by cancerous cells, newly developed CAR-T cells, using a bidirectional promoter for dual CD19 and CD20 targeting, aimed to improve treatment efficacy against lymphoma (105). Interestingly, additional studies are also being ongoing to develop CAR-T cells able to recognize a broad range of tumour-specific antigens and penetrate solid tumours more effectively. For example, the creation of a novel IL13 variant, termed IL13 (4MS) engineered to enhance selectivity for the IL13Rα2 receptor over IL13Rα1, has been incorporated into a bispecific CAR-T design which aimed to improve the efficacy of CAR-T cell therapy against glioblastoma (106).

The cross-reactive toxicities may represent a central limitation in ACT’s clinical application. As a representative example, CAR-T cells targeting HER2 have been reported to cause severe toxicities (i.e. cytokine-release syndrome) due to cross-reactions with healthy tissues (107). To address such toxicities, a cellular safeguard system has been created to selectively eliminate CAR-T cells upon off-target recognition (108). Other useful approach could be the identification of antigens that are exclusively expressed on a particular cancer, like fetal acetylcholine receptors in rhabdomyosarcomas (109). As alternatives, several studies have proposed novel targets like glypican-3 (GPC3) in hepatocellular carcinoma (110) and prostate-specific membrane antigen (PSMA) in prostate cancer (111). In a different way, inhibitory CAR-T cells (iCARs) can co-exist with conventional adoptive T cells but recognizing distinct antigens and transmitting negative signals to reduce off-target effects on healthy tissues (112). Parallelly, to address toxicities related to CAR-T cells long-term overactivation, suicide genes (for example RapaCaspase9 suicide gene system) can be engineered into CAR-T cells, allowing selective elimination upon activation (113). Similarly, SynNotch technology can enhance antigen-specificity and multiantigen targeting of CAR-T cells by post-translationally reprogramming their targeting spectrum. Specifically, this technology can trigger a secondary CAR expression against an additional antigen only upon binding to a first cancer-associated antigen, thus demonstrating potent activity and increased reactivity at lower doses (thus reducing toxicity risks) (114).

Finally, the development of efficient CAR-T cells often requires complex manufacturing procedures that involve virus- or non-virus-based genetic modifications, which increases development costs. To mitigate this, it has been proposed the use of antibody-cell conjugating (ACC) technology to allow the conjugation of T cells with tumour-specific antibodies for targeting cancerous cells without the need of high-cost genetic modifications (115).

On the other hand, it is relevant to briefly note the CAR-based immunotherapies relying on innate immune system. A recent Phase I clinical trial has shown promising outcomes regarding the use of CAR-macrophages against HER2 breast cancer. These CAR-macrophages exerted antitumour activity through several mechanisms like cancerous cells phagocytosis, anti-tumour cytokine release and cancer-specific antigen recognition (116). Specifically, the study demonstrated the manufacturing feasibility, safety and tolerability (absence of cytokine-release syndrome) of this therapeutic approach.

Parallelly, CAR-NK therapy applies CAR engineering to natural killer cells, most commonly using NK cells from cord blood, peripheral blood, or established NK cell lines such as NK-92 (117, 118). Studies have demonstrated several advantages of CAR-NK cells-based immunotherapies compared to traditional CAR-T cells. Among them are included their safer profiles which may allow a wider clinical applicability, certain manufacturing facilities or the simultaneous promotion of both antigen-dependent and antigen-independent antitumour activities. These advantages of CAR-NK cells-based immunotherapies minimize cancerous cells immune evasion related to antigens loss/change (119). Specifically, NK cells are less likely than T lymphocytes to mediate graft-versus-host disease (GVHD), and early clinical data suggest a lower propensity to induce severe cytokine release syndrome (CRS) or immune effector neurotoxicity compared with CAR-T therapies, supporting the development of allogeneic “off-the-shelf” CAR-NK products (118, 120). Their innate cytotoxic receptor repertoire may compensate partially for heterogeneous antigen expression on tumour cells (117, 121). Furthermore, CAR-NKs might offer logistical advantages for allogeneic manufacture, given the possibility of banked donor-or cord blood-derived NK cells (118). Moreover, it has also been suggested the promising therapeutic potential of combining CAR-NK cells with traditional chemotherapy or with own CAR-T cells (119).

For instance, it has also been described the use of CAR-NK cells to eradicate lung cancer cells, exhibiting increased cytotoxicity and cytokine release capacity compared to parental NK cells (122). Recent clinical programmes have tested cord-blood derived CAR-NKs engineered with cytokine support (e.g., IL-15), and early-phase data report encouraging safety profiles and preliminary anti-tumour responses, which has prompted expansion into larger trials (118). Nonetheless, important limitations remain: persistence and *in vivo* expansion of NK products tend to lag behind T cells, which may limit long-term tumour control; clinical data are still far more limited than those for CAR-T (few late-phase randomize trials); and manufacturing standardization of scalable GMP products remains a challenge (e.g., variability between donor-derived NKs, pros/cons of NK-92 lines requiring irradiation vs donor NK expansion) (117–120). Multiple strategies are under development to overcome these hurdles, such as arming CAR-NK with IL-15 or other cytokines to enhance persistence, transient cytokine support, or combining with checkpoint blockade tailored for NK receptors, all aimed to improve durability and potency (118, 121).

### T-cell receptor-engineered T cell therapy

TCR-engineered T cells are autologous or allogeneic T lymphocytes genetically modified to express a defined α/β T-cell receptor with specificity for tumour-derived peptides presented on class I and II MHCs (123, 124). Therefore, TCR-T expands targetability to intracellular neoantigens that are inaccessible to conventional CAR constructs only targeting cell-surface antigens, making TCR-T particularly attractive for solid tumours that lack tumour-specific surface markers but with tumour-specific intracellular peptides (124, 125). Other strengths rely on the use of physiological TCR signalling pathways, which can favour memory formation and nuanced activation, and the minimization of on-target/off-tumour toxicities (124, 126).

Early and mid-phase clinical programs of engineered TCRs directed at NY-ESO-1, MAGE family antigens, and viral antigens have demonstrated objective responses in selected patients with melanoma, synovial sarcoma, and other malignancies (127, 128). For example, a phase I trial of autologous NY-ESO-1-specific TCR-T cells in patients with soft-tissue sarcoma (synovial sarcoma) reported durable tumour shrinkage lasting more than 2 years in one patient, with long-term persistence of the TCR-T cells (127).

Advances in sequencing, neoantigen prediction, and high-throughput TCR discovery have accelerated the pipeline for neoantigen-directed TCR products and have enabled affinity optimisation with extensive safety screening to reduce cross-reactivity (124, 125). Several consortiums and companies have advanced TCR-T programmes into phase II or beyond; comprehensive reviews summarizing clinical outcomes and technical progress have recently been published (129).

On the other hand, critical limitations remain: HLA restriction (a TCR specific for a peptide–HLA allele cannot be broadly applied across HLA types), risk of cross-reactivity to similar peptides in normal tissues (occasionally yielding severe toxicity), tumour immune-evasion through downregulation or loss of antigen-presentation machinery, and the operational/financial burden of autologous manufacturing (124, 129, 130). Some of these limitations may seem in tension with the earlier claim of reduced off-tumour toxicity; indeed, while the physiological signalling may reduce non-specific activation, the risk of on-target but off-tumour reactivity remains non-negligible especially for shared antigens.

Technical strategies proposed to mitigate these issues include exhaustive *in silico* and *in vitro* cross-reactivity screening, development of panels of TCRs covering frequent HLA alleles, and combination strategies to increase antigen presentation (e.g., epigenetic modifiers or IFN-stimulating agents) (124, 129).

The field is trending toward (i) multiplex TCR libraries and off-the-shelf, edited allogeneic TCR products that are HLA-matched or universal; (ii) integration of TCR discovery directly from tumour-infiltrating lymphocytes (TIL) repertoires; and (iii) combining TCR-T with agents that restore or boost antigen presentation (e.g., epigenetic modulators, cytokines) (129, 130).

### Tumour-infiltrating lymphocyte therapy

**Figure 6:**
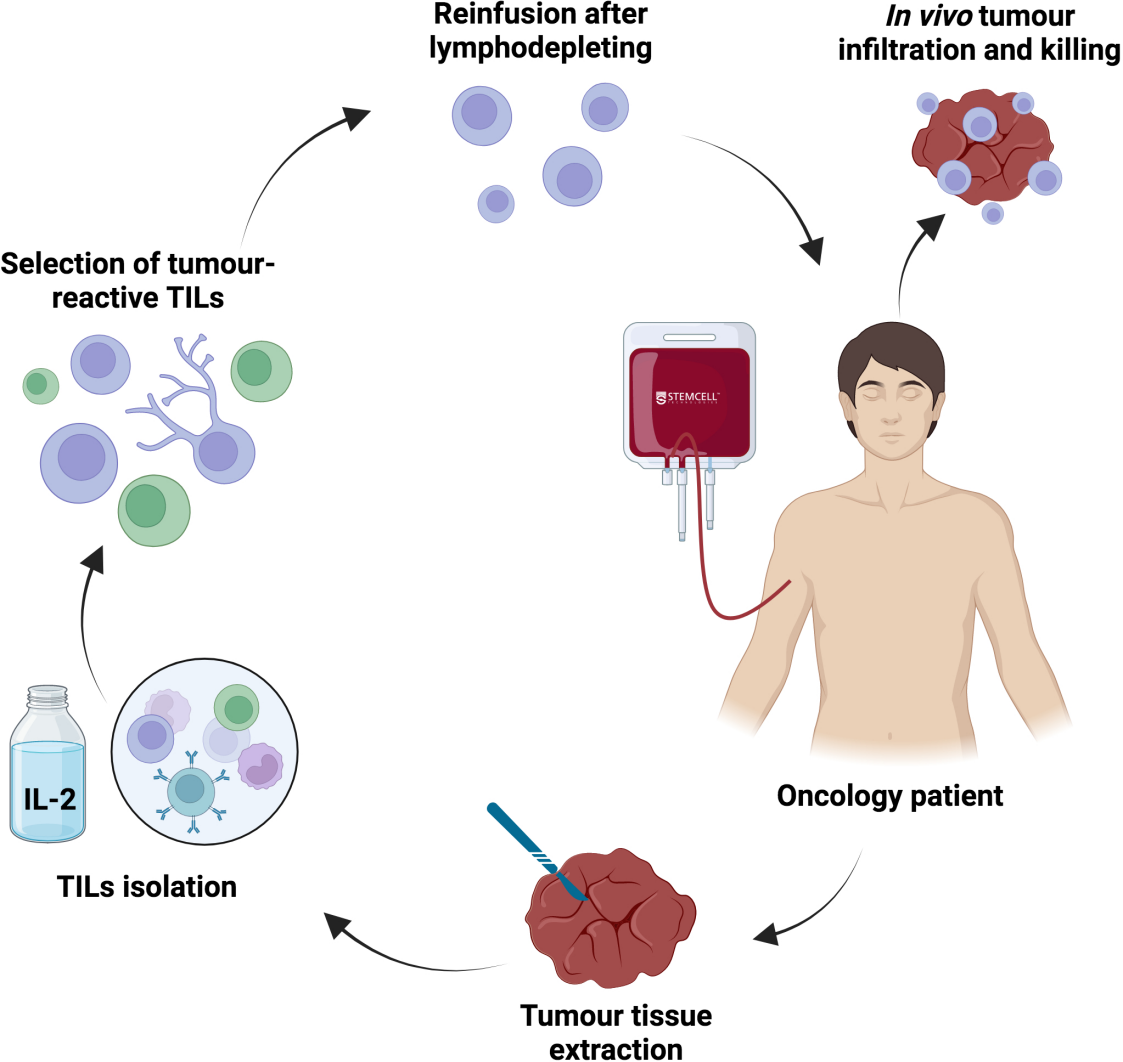
Adoptive T cell therapy: tumour-infiltrating lymphocyte (TIL) therapy. TIL therapy is a personalized adoptive cell therapy that uses naturally occurring tumour-reactive T cells extracted directly from the patient’s tumour. Following surgical resection or biopsy, tumour fragments are processed to isolate resident TILs. These lymphocytes are expanded *ex vivo* using high-dose IL-2, and optionally enriched for tumour-reactive clones. After a lymphodepleting conditioning regimen, the expanded TIL product is reinfused into the patient, where it can recognize multiple endogenous tumour antigens through its native TCR repertoire. Once infused, TILs can proliferate, infiltrate the tumour microenvironment, and mediate direct cytotoxic activity against cancer cells.

TIL therapy relies on the *ex vivo* expansion of polyclonal, tumour-resident lymphocytes obtained from resected tumour tissue, followed by lymphodepletion of the patient and reinfusion of the expanded TIL product (often with systemic interleukin-2 support) (131, 132) (**Figure 6**). This approach leverages a naturally selected repertoire of tumour-reactive T cells, often enriched for neoantigen specificity, and can target multiple tumour epitopes simultaneously, potentially mitigating resistances driven by tumour heterogeneity and antigen loss.

TILs have produced durable responses especially in metastatic melanoma. For instance, in a multicentre Phase II trail of the product Lifileucel in 153 patients with advanced melanoma previously treated with ICIs, the investigator-assessed ORR was 31.4% (8 complete responses ad 40 partial responses), with many responses durable at long-term follow-up (>18 months in a significant fraction of responders) (132). Such data revived interest in TIL-based therapies, even in patients refractory to prior immunotherapy.

Methodological advances have improved the selection and expansion of the most functional clones: for example, use of co-stimulatory agonists (e.g. anti-4-1BB) at the beginning of ex-vivo culture enhances production of CD8-predominant, memory-like TIL with improved effector properties (131). Other strategies under investigation include alternative cytokine support (e.g. IL-7, IL-15), shorter manufacturing protocols, and combination regimens (e.g. preconditioning with oncolytic viruses or radiotherapy) to improve TIL yield and *in-situ* activation (133).

However, broad application is limited by the need for surgically accessible tumour tissue, not all tumours are amenable to safe resection or biopsy yielding enough viable TIL. There is inter-patient variability in TIL yield and quality, and the manufacturing remains resource-intensive. Additional barriers include the substantial toxicity associated with lymphodepletion and high-dose IL-2, the invasive nature of tissue procurement, and lengthy GMP-grade manufacturing times (131, 134). In some non-melanoma solid tumours, especially those with poor lymphocyte infiltration or highly immunosuppressive microenvironments, generation of functional TIL remains challenging (131, 135).

## Cancer vaccines

**Figure 7:**
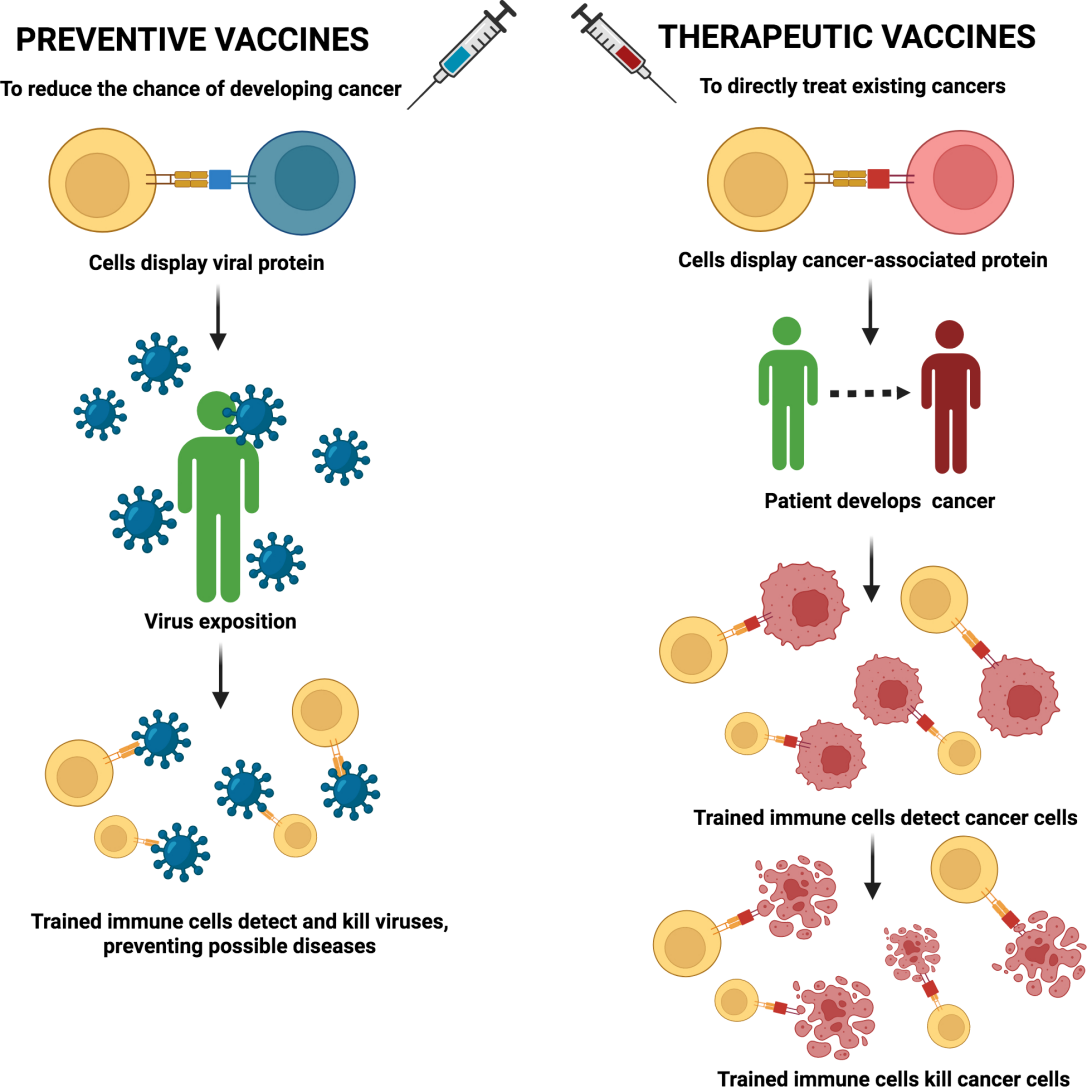
Preventive and therapeutic vaccines’ approach. Preventive vaccines (left) are focused on preparing the body for possible viral agents that could later lead to tumour development. That is, reducing the possibility of developing certain types of cancer. Therapeutic vaccines (right) directly combat cancer once it has already developed in the body. In this case, immune cells recognize a cancer-associated antigen delivered by the vaccine, which allows immune system to detect and directly kill tumour cells.

William B. Coley’s 1891 research pioneered the concept of cancer vaccination by injecting patients with bacterial toxins, leading to tumour regression and laying the groundwork for future cancer vaccines development (1). It may be highlighted two main types of cancer vaccine approaches, first being the preventive vaccination to reduce tumour initiation of cancers that may be triggered by viral infections. As an example, it has been confirmed the durable effectiveness of quadrivalent human papilloma virus vaccines to prevent papilloma virus-related high-grade cervical cancer (136). Secondly, the direct treatment of existing cancers, for instance, the case of the vaccine Sipuleucel-T in the management of metastatic prostate cancer (137). The basis of this therapeutic strategy relies on stimulating the host immune system with external cancer-associated antigens to prevent/combat cancer more efficiently (**Figure 7**).

Cancer vaccines can be designed to stimulate the immune system through various strategies like dendritic cells (DCs)-based vaccines. For example, it has been demonstrated that induced pluripotent stem cells (IPSCs)-derived DCs (iPSC-DCs) can enhance the immune response and potentially overcome resistance to anti-PD-L1 therapy in non-responsive tumour types (138). Similarly, a case report of a psoriatic patient with cutaneous squamous cell carcinoma treated with a combination of radiotherapy, *in situ* vaccination with low-dose autologous DCs and anti-PD-L1 agents enhanced the efficacy of anti-cancer treatment (139). Besides, rWTC-MBTA vaccine has been exposed to effectively and dynamically activate DCs to combat glioblastoma by converting an “immunologically cold” into a “immunologically hot” tumour (140). However, preclinical DC vaccines research may pivot to address clinical failures and practical challenges/issues. These include production difficulties, patient immune variability, testing them against ICI-resistant cancers and boosting T cell activation against “immunologically cold” tumours, in order to enhance its real-world efficacy and accessibility (141). To improve DCs vaccines, it has been shown, for instance, the use of L-fucose (L-fuc) to boosts anti-tumour immunity by enhancing DC polarization, antigen uptake and T-cell activation (142).

An alternative cancer vaccine could be whole tumour cell vaccines (WTCVs) which use inactivated or modified cancer cells with the goal of inducing a host memory response against tumour cells for long-term protection. In fact, it has been emphasized that the strengthening of tumour cells immunogenicity, by enhancing the Irf7 axis within own cancerous cells, can significantly enhance the effectiveness of WTCVs and make them a potent option for preventing tumour recurrence (143). Moreover, it has been investigated the desialylated WTCV (DWTCVs) which can trigger anti-tumour immunity in ovarian cancer patients. Specifically, the process involves treating ovarian cancer cells with enzymes to remove sialic acid residues, thereby exposing antigenic Gal/GalNAc epitopes, increasing antigen uptake by host immune system and increasing T cell activity (144). Another example could be The Aza-BFcell-106 vaccine, which couples Aza-stimulated melanoma cells with a TLR7 agonist and showed enhanced immunogenicity and therapeutic potential in melanoma (145). Nevertheless, WTCVs often fail to halt tumour progression because most tumours commonly exhibit weak immunogenicity, thus several strategies have been reported in order to improve their clinical efficacy. For instance, an advanced oxidation nanoprocessing strategy can enhance the immunogenicity of WTCV, maintaining antigen diversity while ensuring safety and effectiveness (146). Similarly, the Angel-Vax strategy may enhance the host immune response by combining tumour cells with adjuvants polyribocytidylic acid, potentially offering a simpler and more effective option against post-surgical tumour recurrence (147). Furthermore, it has been described the personalized Bridge-Vax vaccine which employs a hydrogel-based platform with autologous tumour cell membranes and that has shown improved efficacy in ovarian cancer by enhancing CD8+ T cells immunity and preventing tumour recurrence (148). Additionally, it has been noted the use of vaccines based on irradiated tumour cells combined with immune-stimulating agents like mannan-BAM, TLR agonists and anti-CD40 antibody to induce an immune response against the tumour mass (149).

**Figure 8:**
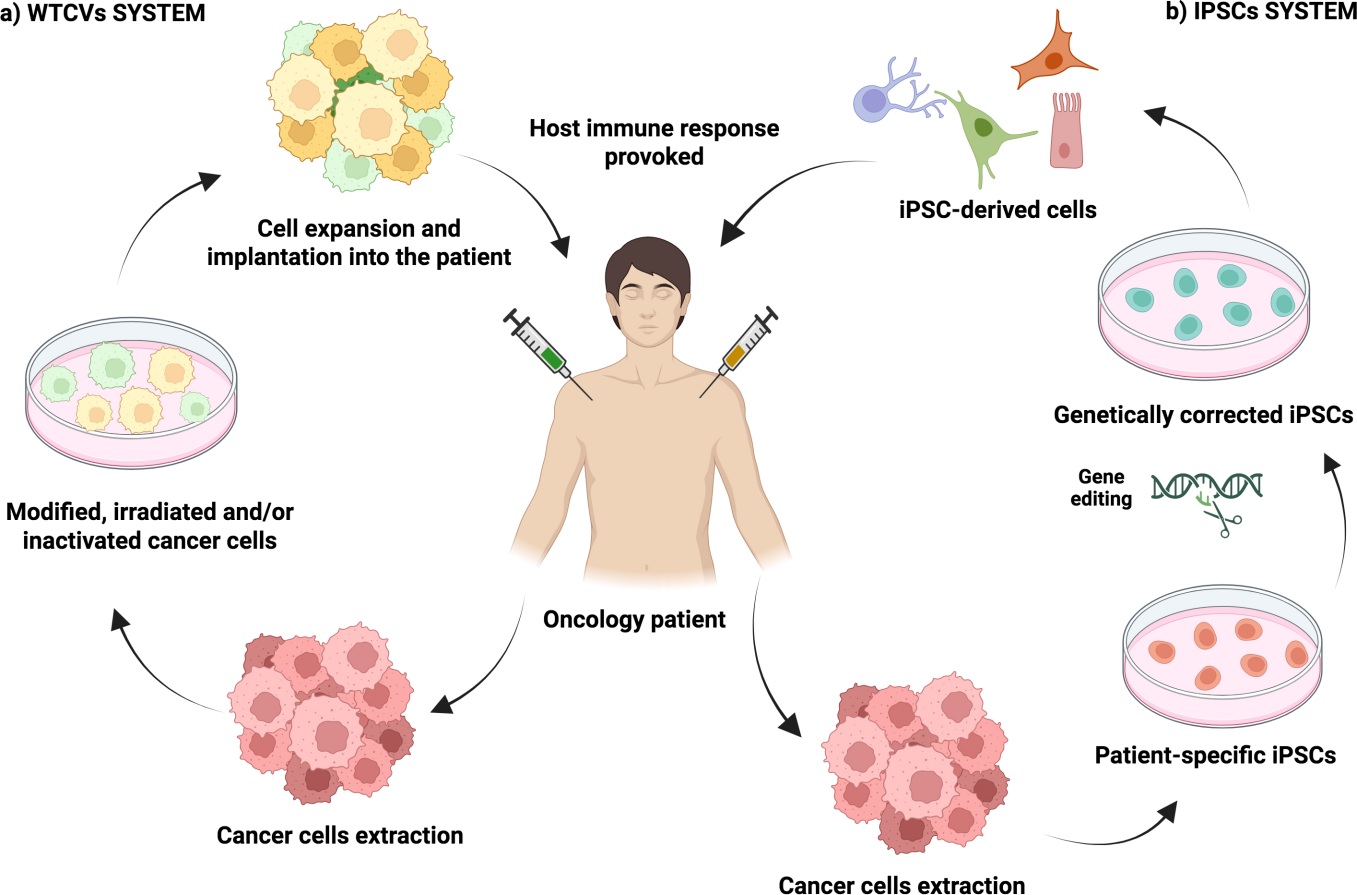
WTCV and iPSCs vaccines. **(A)** WTCV vaccines are based on inactivated, modified and/or irradiated cancer cells to provoke a host long-term immune response. **(B)** Cancerous IPSC-based vaccines are based on the generation of autologous IPSCs derived from host cancer cells and modifying them to reduce their malignancy. They can exhibit a cancer-like antigen profile that can activate host T cells.

Besides, cancerous IPSC-based vaccines rely on the generation of autologous IPSCs derived from host cancerous cells and modifying them to reduce its malignancy. Such cancerous IPSCs can exhibit similar genomic/transcriptomic profiles compared to the own cancer, thus expressing antigens that could activate host T cells. For example, it has been observed promising results from a cancerous iPSC-based vaccine to prevent PC development in mouse models by boosting cytotoxic T cells and stimulating memory-like immune response through targeting antigens/mutations shared between tumours and iPSCs (150). Additionally, it has been described the generation of IPSC-based vaccines from leukemic cells which could inhibit the growth of inoculated tumours in immunocompromised mice without causing significant toxicity (151) (**Figure 8**).

Parallelly, *in situ* vaccines are administered directly into the tumour mass to cause local but not systemic inflammation and immune activation. The rationale is that using an adjuvant immunogenic stimulation may overcome local immunosuppressive TME and activate host immune system against tumour mass. In fact, a study suggested that *in situ* vaccination (ISV) with bacterial outer membrane vesicles is more effective when combined with the use of own tumour neo-epitopes. This strategy can potentially synergize with own cancer cell-derived vaccines to enhance host T cell infiltration and anti-tumour response (152). Another study about ISV against lymphoma achieved a 50% overall response rate, with 70% of patients showing significant tumour reduction outside the treated site (153). In an alternative rationale, viral vaccines use modified viruses to express cancer-associated antigens and trigger a broad host immune response against the tumour. For example, the successful cloning, expression and protein production of the carcinoembryonic antigen gene within adenovirus-based vaccines was characterized by its high efficiency and low toxicity (154). Moreover, in the Phase II trial of the PROSTVAC viral vaccine in prostate cancer patients on active surveillance showed no significant immunologic or clinicopathologic benefits over the control, but confirmed its feasibility and acceptability (155). Therefore, these results may reflect the current limited clinical outcomes of this type of cancer vaccine and the need of further research efforts.

Another related approach may be oncolytic vaccines which use genetically modified viruses to selectively infect and kill cancer cells while sparing normal cells. One example includes Talimogene laherparepvec (T-VEC) for unresectable metastatic melanoma, that showed effectiveness both alone and when combined with ICIs (156). Similarly, another study found that combining pelareorep (reovirus-based therapy) with pembrolizumab and chemotherapy in advanced PC patients was safe and showed promising efficacy (157). Another platform is represented by peptide-based cancer vaccines, which involves the delivery of short peptide sequences (5–15 amino acids) resembling certain cancer- or TME-related antigens epitopes to stimulate a host adaptive immune response against tumours like melanoma (158). Nevertheless, such therapeutic strategy still faces several limitations like relatively low immunogenicity, rapid peptide clearance or insufficient peptide uptake by host antigen-presenting cells (159). Alternative strategies reside on nucleotide-based vaccines (both DNA- and RNA-based ones) encoding cancer-associated neoantigen as observed in a mRNA-vaccine platform capable of inducing long-lasting memory T cells response to combat primary and recurrent pancreatic ductal adenocarcinoma (160). Several issues are being addressed with the aim of optimizing mRNA cancer vaccines, including the modification of RNA sequence to improve its stability for efficient storage, transport, inoculation and/or translation efficiency, along with evaluating alternative delivery strategies to optimize antigen-presenting profiles (161).

Selection of the right cancer vaccine approach is vital to overcome its general limited efficacy in clinical settings, but variability in clinical outcomes and challenges in consistent success still hinder the universal adoption of this cancer immunotherapy (162). Factors like cancer stage, patient age, patient immune system status and particular profile of the immunosuppressive TME play a key role in the diverse outcomes seen across patient groups (163, 164). Cancer vaccines represent a popular researching field in the scientific community and is making significant strides towards their clinical use.

## Conclusions and future perspective

It is emphasized that ICIs have transformed cancer treatment, achieving notable success across various malignancies. However, only around 10-20% of patients achieve durable responses, highlighting a critical area for improvement (165). To optimize the efficacy of ICIs, combination therapies targeting multiple immune evasion mechanisms are increasingly being explored (166). A comprehensive analysis of over 1,000 tumors using CPTAC pan-cancer proteogenomic data has identified seven distinct immune subtypes characterized by unique genomic and proteomic alterations (167). This deep understanding of the immune landscape within tumor tissues not only underscores variations in immune cell surveillance but also reveals potential subtype-specific therapeutic targets, which could pave the way for more effective and personalized immunotherapy strategies in the future. For instance, recent research have indicated that the specific Keap1 mutations can remodel the TME of lung adenocarcinoma, diminishing dendritic cell and T cell responses that drive resistance to immunotherapy (168, 169). Indeed, this study suggests the personalized approach relying on the combination of glutaminase inhibition with ICIs to manage such genetic profile.

Looking ahead, the integration of nanoparticles presents a promising strategy to modulate the TME and enhance the efficacy of ICIs and vaccines. These nanoparticles can effectively counteract TME-related immune evasion by suppressing fibroblast activation, promoting M1 macrophage polarization and facilitating T cell infiltration (170). Additionally, current trends indicate the potential emergence of an “IO (immune-oncology) bubble” in the context of ICIs. This underlines the need for innovative clinical trial designs focusing on new biomarkers, extensive pharmacokinetics and toxicity evaluation, replacement of traditional dose-response trials by dose-ranging ones or the integration of big data/IA analyses. These studies would allow to accurately define the particular genomic/proteomic profiles of each patients in order to develop personalized therapeutic approaches (171–173).

Despite the successes of CAR-T therapy in treating hematological malignancies significant challenges remain in solid tumors (i.e. antigen heterogeneity, immunosuppressive TME, manufacturing costs, treatment-related toxicities), necessitating novel strategies. Among these, CAR ligand-targeted vaccines emerge as a new approach intended to bolster CAR-T effectiveness by enhancing specificity towards tumor-associated antigens. This strategy, along with precision engineering of CAR-T cells using CRISPR-Cas9 and novel genetic modifications, holds promise for improved therapeutic outcomes (174–178). Another innovative approach could be specifically targeting disseminated tumor cells (DTCs) with the goal of significantly reducing the risk of recurrence, metastasis and improve patient outcomes (179). Finally, cancer vaccines encompass a growing range of products with future promise, although addressing patient’s specific host immune profiles and general clinical inefficiency still remain to be improved.

In summary, while cancer immunotherapy offers vast and innovative therapeutic potential compared to conventional treatments, like chemotherapy and radiotherapy, further research is essential to address existing limitations, particularly regarding solid tumors and the establishment of feasible manufacturing protocols (**Table 1** represents an overall summary of main concepts referred to this study). The complexities of the immunosuppressive TME and tumor/patient heterogeneity continue to pose significant challenges, underscoring the need for ongoing exploration in immunotherapy approaches, especially those reliant on the host immune status. **Table 2** summarizes the main advances and limitations described in the present article.

**Table 2:** CAR-T vs vaccine-based immunotherapies vs immune-checkpoint inhibitors (ICIs).

Feature/Axis	CAR-T therapies	Vaccine-based immunotherapies (therapeutic)	Immune checkpoint inhibitors (ICIs)
Mechanism	Genetically modified T cells expressing CARs that bind cell-surface antigens → direct cytotoxicity, cytokine release.	Priming/expansion of tumour-specific T cells (or B cell responses) via antigen presentation; formats include dendritic-cell vaccines (e.g., sipuleucel-T), peptide/protein vaccines, viral vectors, and mRNA neoantigen vaccines.	Antibody blockade of inhibitory receptors (PD-1, PD-L1, CTLA-4) or their ligands to release pre-existing antitumour T-cell responses.
Representative approved/late-stage examples	Tisagenlecleucel, axicabtagene ciloleucel, lisocabtagene maraleucel, idecabtagene vicleucel (BCMA), etc.	Sipuleucel-T (prostate); multiple mRNA neoantigen trials (e.g., mRNA-4157/V940 + pembrolizumab in melanoma; investigational personalized vaccines).	Pembrolizumab, nivolumab, atezolizumab, ipilimumab, durvalumab, etc.
Target space	Surface antigens (CD19, BCMA, HER2, etc.) — limited to proteins on cell membrane.	Intracellular and surface antigens (presented by MHC) — can target neoantigens and tumour-specific peptides.	Release of pre-existing immune responses; reliant on tumour antigenicity and T-cell infiltration.
Typical manufacturing	Autologous leukapheresis → genetic modification (viral or non-viral) → expansion → infusion; complex GMP, weeks.	Variable: autologous cell-based (sipuleucel-T) or off-the-shelf mRNA/peptide vaccines; neoantigen mRNA vaccines require sequencing and custom manufacture (weeks).	Off-the-shelf monoclonal antibodies; standard biologic production.
On/off-the-shelf	Often autologous (patient-specific); allogeneic/UCART in development.	Many vaccine formats are off-the-shelf; personalized neoantigen vaccines are bespoke.	Off-the-shelf.
Clinical niches (strongest evidence)	Haematologic malignancies (B-cell leukemias/lymphomas, multiple myeloma).	Prostate (sipuleucel-T), melanoma adjuvant setting with neoantigen mRNA vaccines (emerging), investigational in multiple solid tumours.	Broad; many tumour types (melanoma, lung, RCC, bladder, etc.).
Efficacy (durability)	High CR rates in some hematologic diseases; durable remissions in responders. Limited success in most solid tumours to date.	Durable immune responses possible; clinical benefit variable — some durable remissions (sipuleucel-T), neoantigen vaccines show promise for recurrence reduction in adjuvant trials.	Durable responses in subset of patients; long-term remissions observed.
Onset of activity	Rapid (days–weeks) after infusion.	Variable — vaccines require weeks to mount T-cell responses.	Variable — responses can occur within weeks to months.
Major toxicities	Cytokine release syndrome (CRS), immune effector cell-associated neurotoxicity (ICANS), on-target/off-tumour toxicity; long-term B cell aplasia for CD19 CARs; secondary malignancy signal under surveillance.	Injection/infusion reactions; variable immune-related adverse events when combined with ICIs; for cell-based vaccines, infusion-related toxicities and cytokines (sipuleucel-T mild).	Immune-related adverse events (colitis, pneumonitis, endocrinopathies, hepatitis), sometimes severe and chronic.
Biomarker needs	Target antigen expression/density, tumour burden, soluble antigen, tumour microenvironment; CAR persistence markers.	Antigen expression/MHC presentation, neoantigen burden/clonality, vaccine immunogenicity readouts (ELISPOT, T-cell repertoire).	PD-L1 expression, tumour mutational burden (TMB), gene-expression signatures, presence of TILs — imperfect predictors.
Resistance mechanisms	Antigen loss/heterogeneity, immune-suppressive TME, poor trafficking to solid tumours, limited persistence.	Poor antigenicity, MHC loss, immune suppression, inadequate APC activation.	Primary/secondary resistance via alternative inhibitory pathways, lack of TILs, tumour intrinsic signalling, compensatory immunosuppression.
Manufacturing/cost & access	Very expensive; complex logistic chain; limited manufacturing capacity currently.	Ranges from low cost (peptide vaccines) to high (personalized mRNA vaccines).	High drug cost but widely distributed manufacturing and administration.
Off-target/safety surveillance	Long-term follow-up required (insertional mutagenesis signals historically; secondary hematologic malignancy warnings posed by regulators).	Safety generally favourable; autoimmunity possible if self-antigens targeted.	Well-established irAE management algorithms; potential for chronic autoimmune sequelae.
Ideal combination partners	ICIs, oncolytic viruses, cytokine support, TME modulating agents, multispecifics.	ICIs, adjuvants, radiation, cytokine modulators; prime-and-boost strategies.	Vaccines, cell therapies, targeted therapy, radiation — to convert “cold” to “hot” tumours.
Regulatory/maturity	Several approved CAR-T products; expansions in indications ongoing; continued post-marketing surveillance.	One long-standing approved autologous vaccine (sipuleucel-T); personalized vaccines advancing rapidly in trials.	Multiple approvals across tumour types; standard of care in many indications.
Clinical trials (Phase I/II/III)	Multiple pivotal and registrational studies across phases II and III for haematologic CAR-T products (e.g., ZUMA-1 for axi-cel; JULIET for tisagenlecleucel; KarMMa for ide-cel; CARTITUDE-1 for cilta-cel with phase 1b/2è confirmatory randomized studies following.	Wide spectrum of trials: long-standing phase III evidence for autologous celular vaccine sipuleucel-T (IMPACT trial); randomized phase II (2b) positive data for individualized mRNA neoantigen vaccine mRNA-4157/V940 + pembrolizumab (KEYNOTE-942) with ongoing phase III programmes initiated/planned in melanoma and other indications. Multiple early-phase (I/II) trials of peptide, viral-vector and personalized neoantigen vaccines are active across tumor types.	Large number of phase III randomized trials establishing efficacy in multiple tumor types (examples: KEYNOTE series for pembrolizumab – including KEYNOTE-189 in NSCLC first-line; numerous phase III trials underpin approvals of nivolumab, atezolizumab, durvalumab, ipilimumab, etc.). Ongoing combination and adjuvant/neoadjuvant phase II/III trialscontinue to expand indications.
Representative references	(180–185)	(186–190)	(191–195)

## Glossary

ACC: Antibody-Cell Conjugating
ACT: Adoptive T Cell Therapy
ALL: Acute Lymphoblastic Leukemia
ATM: Ataxia-Telangiectasia Mutated
BSABs: Bi-specific Antibodies
CAR: Chimeric Antigen Receptor
CD: Activation Domain
CRS: Cytokine Release Syndrome
CTLA-4: Cytotoxic T-Lymphocyte-Associated Protein 4
DCs: Dendritic Cells
DLBCL: Diffuse Large B-cell Lymphoma
DLD: Dihydrolipoyl Dehydrogenase
DNA: Deoxyribonucleic Acid
DTCs: Disseminated Tumour Cells
DWTCV: Desialylated Whole Tumour Cell Vaccines
FAP: Fibroblast Activation Protein
GPC3: Glypican-3
GVHD: Graft-versus-hot Disease
HDAC: Histone Deacetylase
iCARs: Inhibitory CAR-T Cells
ICIs: Immune Checkpoint Inhibitors
IH: Immune-related Hypophysitis
IO: Immuno-Oncology
IPSCs: Induced Pluripotent Stem Cells
IRAEs: Immune-related Adverse Events
ISV: *In Situ* Vaccination
L-fuc: L-fucose
LAG-3: Lymphocyte Activation Gene-3
MDSCs: Myeloid-derived Suppressor Cells
MHCs: Major Histocompatibility Complexes
MSI: Microsatellite Instability
NK: Natural Killer
PC: Pancreatic Cancer
PD-1: Programmed Death-1
PSMA: Prostate-specific Membrane Antigen
RNA: Ribonucleic Acid
scFv: Single-chain Variable Fragment
T-Vec: Talimogene laherparepvec
TCR: T Cell Receptor
TIL: Tumour-infiltrating Lymphocytes
TLRs: Toll-like Receptors
TMB: Tumour Mutational Burden
TME: Tumour Microenvironment
Treg: Regulatory T Cells
TSABs: Tri-specific Antibodies
WTCVs: Whole Tumour Cell Vaccines
